# Bacteriospermia and Sperm Quality of Cryopreserved Bull Semen Used in Artificial Insemination of Cows in South Wollo Zone, Ethiopia

**DOI:** 10.1155/2020/2098315

**Published:** 2020-01-23

**Authors:** Abadi Amare Reda, Gizat Almaw, Solomon Abreha, Wedajo Tadeg, Belege Tadesse

**Affiliations:** ^1^School of Veterinary Medicine, Wollo University, Dessie, Ethiopia; ^2^Department of Microbiology, National Animal Health Diagnosis and Investigation Centre, Sebeta, Ethiopia; ^3^Department of Animal Science, College of Agriculture, Wollo University, Dessie, Ethiopia; ^4^Kombolcha College of Agriculture, Kombolcha, Ethiopia

## Abstract

The objectives of this trial were to estimate prevalence of bacteriospermia, to determine the bacterial load, and to isolate the types of bacteria as well as to assess the association between bacterial load and sperm quality traits in cryopreserved bull semen in field conditions in the South Wollo Zone. A total of 309 cryopreserved straws of semen from the Holstein Friesian (HF)-cross bull (*n* = 180 straws) and pure Jersey bull (*n* = 129 straws) were investigated. Bacteriological assessments of the presence of aerobic bacteria, estimation of bacterial count and bacterial isolation, as well as semen quality were performed. Aerobic bacterial contamination was prevalent in 38.8% of the semen straws. No significant difference in the prevalence of bacteriospermia was observed among bulls although the HF-cross bull had a higher prevalence (40.0%). But, significant difference in prevalence of bacteriospermia was found among semen ejaculates of the same bull. The risk of bacteriospermia in the HF-cross bull was higher (Odds ratio = 1.86, 95% CI = 0.168–20.26) compared to Jersey although not significant. Overall average bacterial load of 50.38 ± 16.29 colony-forming units (CFU)/ml (from nil to 1318.20 CFU/ml) was found. No significant difference in bacterial count among bulls and their ejaculates was observed. Moreover, correlation analysis revealed that the proportions of motility, live, and normal morphology were negatively influenced by an increase in the bacterial contamination of semen. In this study, three isolates of coagualse-negative Staphylococcus species and one isolate of Corynebacterium species were found. Average percentages of sperm motility (48.35 ± 1.23), live (66.08 ± 1.0), and normal morphology (80.62 ± 1.24) were observed. It was concluded that cryopreservation does not guarantee the quality of semen from bacterial contamination. Hence, meticulous care should be adopted to prevent contamination of semen by bacteria during collection, transportation, processing, and storage times.

## 1. Introduction

Ethiopia inhabits significant proportion of cattle population in Africa with its 56.71 million heads of cattle [1]. Indigenous cattle are the main livestock species used for dairy production in Ethiopia, which contributes around 81.2% of the total national annual milk yield [2]. Although the country has a huge potential for milk production, the genetic potential and productivity of local breeds, as a matter of fact, is very low [3]. Consequently, the direct contribution of the dairy sector to the national economy is inadequate [1]. More recently, the country has designed a dairy cattle improvement plan to enhance productivity of local breeds through genetic selection, breeding, and artificial insemination (AI) programs [4]. So as to realize this, accurate prediction of bull fertility is essential because it determines the economic success and sustainability of the dairy industry. This in turn is reliant on obtaining high conception rates after insemination of cows using frozen-thawed semen [5].

AI is the oldest and most popular assisted reproductive biotechnology allowing the use of genetically superior males from elsewhere for genetic improvement of livestock [6]. Long-term storage of semen in liquid nitrogen (−196°C), through cryopreservation, is indispensable in order to realize many of the potential advantages of AI. Cryopreservation halts the metabolic activities of spermatozoa, which allows unlimited storage without substantial loss of fertility. Hence, quality of frozen-thawed semen is an important factor that predicts bull fertility and the sustainability of AI as well as investment of bulls with high genetic merits [7]. However, quality of semen can be altered, among others, by the process of cryopreservation [8–13], types of extender used [14], and bacterial contamination [15–18].

Prevalence of bacteria within sperm “bacteriospermia” had detrimental effects on sperm cell function by reducing sperm motility, viability, and abnormal morphology as well as premature acrosome reaction. Other ways by which bacteriospermia affects fertility is by altered mitochondrial function, which provokes formation of reactive oxygen species thereby increasing DNA fragmentation [15, 19]. These effects of bacteria have been reported to negatively impact the viability and fertility of spermatozoa, thereby resulting in total breeding failure. Thus, one of the main factors contributing to AI failure is the contamination of the germplasm with pathogens, which eventually cause loss of fertility [20]. Most common microorganisms that contaminated bull semen were from species of Corynebacterium, Staphylococcus, Micrococcus, Bacillus, Escherichia, Proteus, Pseudomonas, Klebsiella, Streptococcus, Citrobacter, Enterobacter, and Stenotrophomonas [21–25]. These pathogens, in one way or other, may infect inseminated females or contribute to a rapid deterioration in sperm quality [26]. Nevertheless, scientific evidence based on published data concerning the major risk pathogens or bacterial contamination of the germplasms used in AI operations in Ethiopia has been lacking until recently.

In Ethiopia, AI has been applied for over five decades to crossbred indigenous cattle with bulls of known genetic merits imported from different countries. Although AI in Ethiopia has a long history, the success rate of AI in Ethiopia has been and is generally poor. The National AI Centre (NAIC) is the chief source of semen and other consumables for the procedures of AI throughout the whole country. However, many constraints have been reported associated with the source, the selection procedures, and health status of the AI bulls at NAIC [27]. This was further revealed by the unsatisfactory success of AI in regional and district AI centres due to different factors. The major constraints have been linked to lack of infrastructure, lack of AI technician's experience, or due to heat detection problems, management problems, and disease problems [28, 29]. These factors coupled with environmental causes, improper handling, transportation, and storage of semen that deteriorates semen quality including motility, thereby affecting subsequent fertility [30]. Nevertheless, the effects of microbiological contaminants such as bacteria in bull (fresh or frozen) semen have not been assessed in Ethiopia, yet. Thus, this research explores the prevalence of bacteriospermia and semen quality of cryopreserved semen of bulls used in AI of cows in the South Wollo Zone, Ethiopia. Moreover, bacterial load and the types of bacterial contaminants of cryopreserved semen were investigated for the first time in Ethiopia.

## 2. Materials and Methods

### 2.1. Description of Study Area

**Figure 1:**
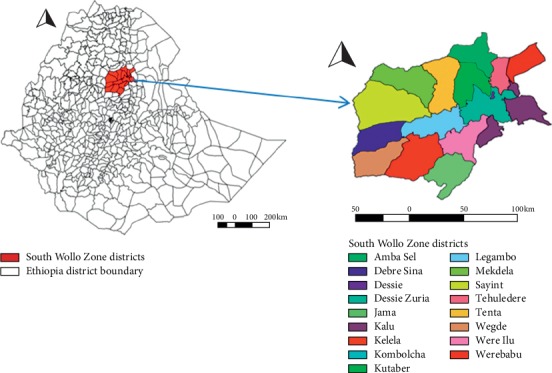
Map of South Wollo Zone Districts.

For this study, cryopreserved French ministraws of bull semen were purchased from South Wollo Zonal Liquid Nitrogen Production and Semen Distribution Centre (SWLNPSDC), Dessie, Ethiopia. Dessie, the largest city in South Wollo Zone of the Amhara Region, is located in the north central part of the country at a distance of 401 km north of Addis Ababa. This city is placed at latitude of 11′8°N and longitude of 39′38°E with an altitude range of 2470 to 2550 meters above sea level. The area has an average annual rainfall of 1145 mm and a mean annual temperature of 15.2°C. A combination of crop and livestock production is the main farming system of the precinct. The total cattle population of the district was estimated at 1.75 million cattle [1]. Both natural service and artificial insemination (AI) are used for crossbreeding of dairy cows for genetic improvement of local breeds despite its low achievement. Semen and other accessories for AI is obtained from the NAIC, Kality, Ethiopia, and stored in the SWLNPSDC. Semen and liquid nitrogen from SWLNPSDC for AI are dispatched to the district AI centres located in South Wollo Zone (Figure 1).

### 2.2. Study Bulls and Semen

A total of 309 cryopreserved French ministraws (0.25 ml) of semen were purchased from SWLNPSDC for this study. These straws was initially brought from NAIC, Kality, Addis Ababa, and stored at the SWLNPSDC for use in insemination of dairy cows found in the area. Of 309 straws, 180 and 129 semen straws, respectively, were collected from two breeding bulls of Holstein Friesian (HF)-cross and Jersey stud at NAIC. The age of HF-cross and Jersey bulls was 60 and 17 months, respectively, and all bulls were healthy. Straws were randomly picked from different ejaculates of these bulls. Each straw had labeled for breed of bull and date of semen production. Date of production was used to calculate the length of storage time, which was defined as the period from production until the sample was processed in the laboratory. The storage period of semen (cryopreservation) for each ejaculates (*n* = 6) was ranged from 300 to 370 days. Individual straws from different ejaculates were considered as sampling units where percentages of semen quality traits and bacterial count were considered as variables.

### 2.3. Study Design and Methodology

Cross-sectional study with simple random sampling technique was carried out from September 2015 to August 2016 to determine the bacterial contamination of cryopreserved bull semen straws stored at the SWLNPSDC. Study methodology was based on the laboratory analysis of semen characteristics (proportions of motility, live, and normal morphology), bacterial count, and isolation.

### 2.4. Semen Characteristics

A total of 103 straws were assessed for the dynamics of semen characteristics (proportions of motility, live, and normal morphology) at the laboratory of NAIC. Estimation of the percentage of the individual sperm motility, live, and normal morphology of cryopreserved semen was done by well-experienced examiner in the Centre. Sperm motility was assessed according to the method reported by [31]. In brief, the straws were thawed at 37°C for 30 seconds in a water bath, and a drop of thawed semen was placed over a warm slide and covered by cover slide. Phase contrast microscope with a magnification of 200X was used. Several fields were examined to estimate the percentage of individual sperm motility. Furthermore, assessment of proportions of live and normal morphology was performed using an eosin-nigrosin staining technique and wet smear method using formal saline, respectively, as described previously [32, 33].

### 2.5. Bacterial Count

A total of 206 straws, which was pooled into 103 straws (2 × 103 straws), were examined for bacterial count and bacterial isolation at the National Animal Health Diagnosis and Investigation Centre (NAHDIC), Sebeta, Ethiopia. Bacterial contamination load was determined following the work of [34]. Accordingly, two straws (2 × 0.25 ml) were mixed and 0.1 ml of it was added in duplicate to sterile Petri dish for count as the original sample. Also, from this sample, 0.1 ml diluted semen was added to 0.9 ml normal saline solution step by step to give serial dilutions of 1 : 10, 1 : 100, 1 : 1000, and 1 : 10000. The same to original sample from each dilution 0.1 ml was poured into duplicate sterile Petri dishes after homogenized with vortex. To each plate, 12–15 ml plate count agar (Oxoid: CM0325) was poured, and thereafter, the sample and agar medium was mixed thoroughly and uniformly. A control for the saline and environment was also prepared. The agar was let to solidify, inverted, and incubated aerobically for 48 hours at 32°C. Counting was conducted with an electronic colony counter. Bacterial load of aerobic plate counts (APCs) was calculated [35], and the result was expressed as colony-forming units per milliliter (CFU/ml) of semen using the following formula:

(1) $$\begin{array}{c} N=\frac{\sum c}{\left[\left(1\times n_{1}\right)+\left(0.1\times n_{2}\right)\right]\times \left(d\right)}, \end{array}$$

where *N* = number of colonies per ml or g of product, ∑*c* = sum of all colonies on all plates counted, *n*_1_ = number of plates in the first dilution counted, *n*_2_ = number of plates in the second dilution counted, and *d* = dilution from which the first counts were obtained.

### 2.6. Aerobic Bacterial Isolation

Isolation was conducted following standard procedures described by [36]. A loopful of semen used for count was cultured on blood (Oxoid: CM00558) and MacConkey agar (Oxoid: CM0007) at the same time and incubated at 37°C for 24 hours. Growth characteristics were recorded including haemolysis on blood agar and lactose fermentation on MacConkey. Primary tests including Gram's reaction, catalase, oxidase, oxidation-fermentation (O-F), motility, glucose, and growth on blood and MacConkey agar were conducted. The coagulase test was also conducted on Staphylococcus isolates.

### 2.7. Statistical Model and Analysis

The bacterial count (CFU/ml) was converted to the natural logarithmic, whereas percentages of sperm motility, live, and normal morphology of spermatozoa were arcsine transformed. However, for ease of interpretation, untransformed data were statistically computed, but linear regression of transformed data was also computed for comparison with the untransformed data.

Data generated from this study were expressed as mean ± SEM of semen motility, live, and normal morphology and bacterial load (CFU/ml) of semen. The statistical analysis was done by applying the general linear model using SPSS computer software package (Version 20.0. for Windows, SPSS Inc., Chicago, IL, USA) [37]. Analysis of variance (ANOVA) was computed to compare mean of semen characteristics between breed, ejaculates, and storage time. Pearson correlation coefficient was performed to measure the strength of linear association between percentages of semen characteristics and bacterial count. Logistic regression was also performed to determine the prevalence of bacteriospermia and the odds ratios among breed and different ejaculates.

## 3. Results

### 3.1. Cryopreserved Semen Characteristics among Bulls and Their Ejaculates

The difference in mean percentages of semen parameters evaluated among HF-cross and purebred Jersey bulls is given in Table 1. In the present study, percentage of sperm motility (±SEM) of cryopreserved semen of HF-cross and purebred Jersey bulls was 51.56 ± 1.58% and 43.69 ± 1.75%, respectively, with a total average motility of 48.35 ± 1.23%, as depicted in Table 1. A total percentage of live spermatozoa and normal spermatozoa was 66.08 ± 1.01 and 80.62 ± 1.24, respectively. Significant (*p* < 0.01) differences were found in the proportion of sperm motility among bulls. Higher percentage of sperm motility was recorded in the HF-cross bull compared to the purebred Jersey bull. No significant difference was observed in the percentages of live and normal spermatozoa among the bulls. In addition, semen motility was significantly (*p* < 0.01) varied among different ejaculates of the same bulls. However, there was no significant difference in the proportions of live spermatozoa and normal spermatozoa between ejaculates of the same bulls, as shown in Table 2.

### 3.2. Bacterial Load of Cryopreserved Semen among Bulls and Their Ejaculates

**Figure 2:**
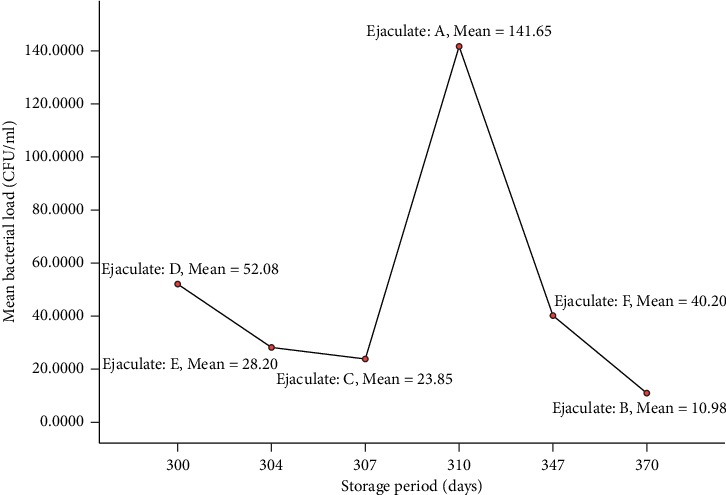
Line plots of mean bacterial load (CFU/ml) over different periods (days) of cryopreservation.

In the present study, the bacterial load of cryopreserved semen was assessed and the total bacterial mean count was 50.38 ± 16.29 CFU/ml. No significant difference was observed in the mean bacterial count among bulls. Jersey bulls had a higher bacterial mean count of 69.05 ± 32.96 CFU/ml compared to HF-cross (37.52 ± 15.62 CFU/ml), as shown in Table 3. Similarly, the mean bacterial count was not significantly varied among the different ejaculates of the same bull (*p* ≥ 0.05), as presented in Table 4. Average bacterial load from two ejaculates of Jersey bull semen was ranged from 10.98 ± 8.32 to 141.65 ± 69.50 CFU/ml. The bacterial load from four ejaculates of HF-cross bull semen was between 23.85 ± 11.32 and 52.08 ± 37.77 CFU/ml. Ejaculates from Jersey bulls had a higher bacterial contamination during 310 days of storage under cryopreservation, as depicted in Figure 2.

### 3.3. Linear Regression and Pearson Correlation among Bacterial Load and Semen Traits

**Figure 3:**
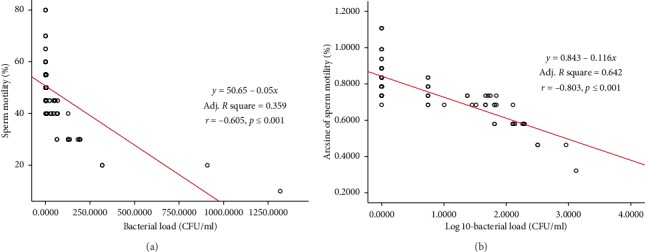
Scatter plot distribution of individual observations for bacterial load and semen motility: (a) untransformed data; (b) transformed data. Linear regression equation, adjusted *R*^2^, and Pearson correlation estimates are also depicted.

**Figure 4:**
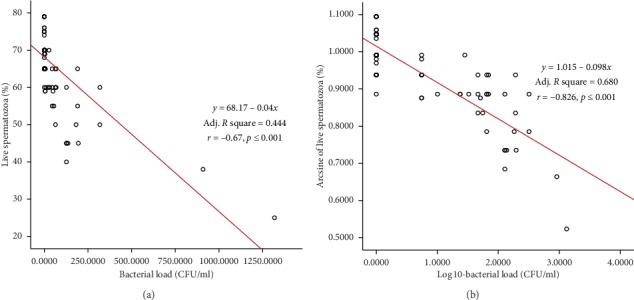
Scatter plot distribution of individual observations for bacterial load and live spermatozoa: (a) untransformed data; (b) transformed data. Linear regression equation, adjusted *R*^2^, and Pearson correlation estimates are also depicted.

**Figure 5:**
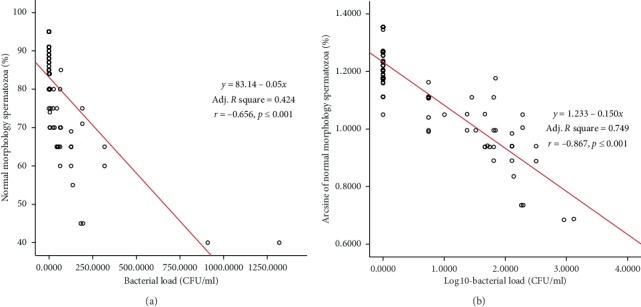
Scatter plot distribution of individual observations for bacterial load and normal spermatozoa: (a) untransformed data; (b) transformed data. Linear regression equation, adjusted *R*^2^, and Pearson correlation estimates are also depicted.

The linear regression and Pearson correlation estimates between bacterial load and semen characteristics (untransformed and transformed data) are depicted in Figures 3–5. Significant (*p* < 0.01) negative association was observed between bacterial load and semen motility% (*r* = −0.605), live% (*r* = −0.670), and normal morphology% (*r* = −0.656), as shown in Figures 3–5. Semen motility was positively correlated with live% (*r* = 0.852, *p* < 0.001, *R*^2^ = 0.727) and normal morphology (*r* = 0.836, *p* < 0.001, *R*^2^ = 0.699). Very strong correlation was observed between live% and normal morphology (*r* = 0. 914, *p* < 0.001, *R*^2^ = 0.835). Multiple linear regression analysis revealed that bull fertility indicators such as the proportions of motility, live, and normal spermatozoa were negatively influenced by an increase in the bacterial contamination of semen (Table 5). On the contrary, regression analysis among the semen parameters indicated that bull fertility could be accurately (*R*^2^ range = 0.699–0.835) predicted by either of the semen quality indicators.

### 3.4. Bacteriospermia and Types of Bacterial Isolates

In the present study, from the total of 103 straws, 40 straws had aerobic bacteria. Hence, the prevalence of bacteriospermia was 38.8% (Table 6). Nonsignificant discrepancy (*p* ≥ 0.05) was observed in prevalence of bacteriospermia among bulls although HF-cross bull had a higher prevalence (40.0%) compared to Jersey (37.2%). But, significant difference (*p* < 0.5) in prevalence of bacteriospermia was found between ejaculates of the same bull (Table 6). Logistic regression analysis (Table 7) showed that the risk of bacteriospermia in the HF-cross bull was increasing by 1.86-fold compared to the Jersey bull although not significant. Moreover, the risk of bacteriospermia was significantly decreased (odds ratio = 0.07, *p*=0.001) in ejaculate B compared to ejaculate A (Table 7).

Of the 40 straws which had aerobic bacteria, two different species of bacteria were isolated. A total of four bacterial isolates (10%) were found, in which three isolates were coagualse-negative *Staphylococcus* (CoNS) species (7.5%) and one isolate was Corynebacterium species (2.5%). Corynebacterium species was found only in straws of the HF-cross bull, whereas coagulase-negative Staphylococcus species was isolated from both Jersey and HF-cross bulls.

## 4. Discussion

Comprehensive assessment of bull fertility [5] as well as microbiological checkup of cryopreserved semen beforehand is of paramount significance for successful AI [20]. The present study describes the prevalence of bacteriospermia, bacterial load, bacterial isolates, and its consequences on sperm functional characteristics in cryopreserved bull semen. The findings demonstrate that aerobic bacterial contamination in 38.8% of cryopreserved semen straws originated from two representative breeding bulls. HF-cross bull had a higher prevalence (40.0%) compared to Jersey (37.2%) although not statistically significant. But, significant difference in prevalence of bacteriospermia was found among semen ejaculates of the same bull. The risk of bacteriospermia in the HF-cross bull was increasing by 1.86 fold compared to the Jersey bull although not significant. Prevalence of bacteriospermia found in this study is almost incongruent with [23, 38], who recorded 40% and 45%, respectively, in frozen semen of cattle. This was higher than the prevalence rate of 7% [39] and 20% [25], which was observed in frozen semen of cattle. However, the current prevalence of bacteriospermia was lower compared to the report of [40], who publicized about 50% in cryopreserved bull semen samples. Moreover, a higher prevalence of bacteriospermia, for instance, 97% [41] and 99% [42] in ram semen and 63% [43] in boar semen, was reported. On the contrary, a lower prevalence comparable to current results, 33.2% [44] and 35.3% [45], was reported in human semen samples. This difference in bacterial contamination of semen may be due to differences in species, age, breed, season [17, 46], anatomy, physiology, and management [41]. It may also be ascribed to unsanitary conditions during semen collection, transportation, and processing or resistance to antibiotics used in semen extender [25].

Microorganisms that contaminate semen can emanate from several biotic and abiotic sources [47]. Biotic sources include normal flora of prepuce, skin, urogenital passageways, hair, respiratory fluids, and feces, poor sanitary conditions, as well as contaminations related to laboratory technicians [48, 49]. The potential abiotic contaminants could come from laboratory environments such as semen collection, transporting and processing equipment, processing laboratory, glassware, buffers, extender, and straws [50]. Other contaminants are related to semen diluents like egg yolk [14, 51], resistant to antibiotics [52], and low efficacy of antimicrobials used in the extender [16, 53–55]. Nevertheless, bacterial load can be reduced considerably by applying hygienic practices of packaging, handling, sterilization and fumigation protocol [48, 50], and preputial washing [56].

Semen should fulfill quality standards of motility, viability, and morphology as well as devoid of microorganisms or minimum load of bacteria before use for AI [54]. The present study revealed that an overall average bacterial load of 50.38 ± 16.29 CFU/ml with a minimum of nil and maximum load of 1318.20 CFU/ml. No significant discrepancy in bacterial count among HF-crossbred and Jersey bulls and their ejaculates was observed. Moreover, correlation and regression analysis revealed that motility%, live%, and normal morphology% were negatively influenced by an increase in the bacterial contamination of semen. Our findings are virtually supported by Shukla [57], who found a mean bacterial load of 48.60 ± 0.73 bacteria/ml in cryopreserved Buffalo bull semen. There was also a highly significant negative correlation of bacterial count with percentages of sperm motility and live sperm in cryopreserved semen. This was further illustrated by positive association observed among dead and abnormal spermatozoa with bacterial count [57], which is in agreement with the present study. Bacterial load obtained in this study may be virtually compared to [16, 40, 58]. They found 1.1 × 10^3^ CFU per ml in crossbred cattle, 1.1 × 10^2^ CFU per ml in crossbred bulls, and 0.07 × 10^4^ CFU per ml in buffalo, respectively.

The bacterial count recorded in this study is higher than in [23], in which a bacterial load of 10.43–12.49 CFU/ml was reported in imported frozen semen of cattle. However, significantly higher bacterial counts of 8.1–39.04 × 10^3^ per ml were documented in frozen semen of Holstein [59] compared to the results found in this study. This variation may be due to differences in adhering strict sanitary and hygienic circumstances during semen collection, processing, and storage under cryopreservation. The relatively good quality of cryopreserved semen observed in the present study (average motility% = 48.35 ± 1.23, live% = 66.08 ± 1.0, and normal morphology% = 80.62 ± 1.24) may be due to low bacterial contamination. Significant negative association of bacterial count and sperm functional markers have been reported [57, 60].

Of the 40 semen straws which had aerobic bacteria, a total of four bacterial isolates (10%) were found in this study, in which three isolates were coagulase-negative Staphylococcus (CoNS) species and one isolate was Corynebacterium species, which was isolated for the first time in Ethiopia. These bacteria along with other species of Bacillus, Micrococcus, Escherichia, Actinobacteria, Flavobacterium, Proteus, Pseudomonas, and Streptococcus have also been isolated as potential contaminants of bovine semen [15, 21, 25, 39]. The identification of most of these bacteria has been correlated with reproductive disorders such as repeat breeding, abortion, and infertility in cattle [25]. In other species, *Staphylococcus epidermidis* in boar semen [43] and CoNS in human semen samples [45] were identified. The discrepancy found in the present study when compared to the above authors may be due to differences in sample size, bacteriological techniques [24], and age and breeds of animals [17].

*Staphylococcus epidermidis* and *Staphylococcus saprophyticus* are commonly known species of CoNS that frequently causes opportunistic contaminations in humans and sometimes in animals [36]. Amongst the Corynebacterium species, *Corynebacterium renale*, *Corynebacterium pilosum*, and *Corynebacterium cystitidis*, which occupy the genital tract, prepuce, semen, and urine of bulls, usually cause pyelonephritis and cystitis in cattle [36]. Motility and viability were significantly declined when human sperm was infected *in vitro* by *Staphylococcus epidermidis*, which is among the CoNS. Hence, it was deduced that detrimental consequences in fertility was associated with prevalence of bacteria in semen [61]. Generally, the prevalence of these bacteria is noteworthy as they may adhere to spermatozoa and decrease sperm motility [62–65]. They can also damage acrosomes by producing toxin [66] and cause DNA damage [15] by producing reactive oxygen species [66]. Eventually, this could result in decreased fertility rates and culling of breeding animals and thus bring about considerable financial losses to dairy industry [67]. Hence, it is imperative to meticulously preclude the microbial contaminants from entering semen processing and storage facilities and AI operations [68].

## 5. Conclusions

The results of the present research showed that substantial proportion of semen straws was contaminated with aerobic bacteria (from nil to 1318.20 CFU/ml). The difference in the prevalence of bacteriospermia among bulls was not significant, but significant difference was found among semen ejaculates of the same bull. No significant difference in the risk of bacteriospermia among bulls was observed despite the risk of bacteriospermia in the HF-cross bull was higher compared to Jersey. This finding suggests that the presence of other sources of semen contaminants in addition to the intrinsic/extrinsic sources related to the host. This may include semen collection, transporting and processing equipment, processing laboratory, laboratory technician, glassware, buffers, semen diluents/extender, straws, and resistance to antibiotics. The relatively good quality of cryopreserved semen observed in the present study may be due to low bacterial load found. Nevertheless, the proportions of motility, live, and normal morphology were observed to be negatively influenced by an increase in the bacterial contamination of semen. In this study, three isolates of coagualse-negative Staphylococcus species and one isolate of Corynebacterium species were found. Since the sample size related to number of bulls, breed, and ejaculates was few, isolation of anaerobic bacterial species as well as antimicrobial susceptibility of the aforementioned bacterial isolates was not performed in this study; further research is recommended. Generally, the present research heralded that cryopreservation does not guarantee the quality of semen from bacterial contamination. This necessitates meticulous sanitary and hygienic measures to prevent contamination of semen by bacteria during collection, transportation, processing, and storage times. Hence, this research may provide good insights into designing appropriate sanitary and hygienic protocols to preclude bacterial contamination of semen samples in AI centres.

## Figures and Tables

**Table 1:** One-way ANOVA of semen characteristics of breeding bulls (no. of straws from Jersey = 43 and HF-cross = 60).

Semen parameters	Breed	Mean ± SEM	95% CI	*F*	*p* value
Motility (%)	Jersey	43.69 ± 1.75	40.16–47.22	10.81	0.001
	HF-cross	51.56 ± 1.58	48.40–54.72		
	Total	48.35 ± 1.23	45.91–50.79		

Live (%)	Jersey	65.45 ± 1.61	62.19–68.71	0.26	0.609
	HF-cross	66.51 ± 1.30	63.91–69.11		
	Total	66.08 ± 1.01	64.08–68.08		

Normal morphology (%)	Jersey	81.57 ± 1.65	78.24–84.91	0.40	0.529
	HF-cross	79.97 ± 1.77	76.42–83.51		
	Total	80.62 ± 1.24	78.16–83.09		

SEM = standard error of mean; CI = confidence interval.

**Table 2:** One-way ANOVA of % sperm motility, viability, and morphology from different ejaculates of Jersey and HF-cross bulls.

Ejaculates	No. of straws	Motility (%)		Live (%)		Normal morphology (%)	
		Mean ± SEM	95% CI	Mean ± SEM	95% CI	Mean ± SEM	95% CI
A	19	37.63 ± 2.90	31.55–43.72	60.47 ± 3.02	54.12–66.83	74.58 ± 2.92	68.44–80.72
B	24	48.54 ± 1.43	45.59–51.49	68.96 ± 1.13	66.63–71.29	86.42 ± 1.00	84.34–88.49
C	21	53.33 ± 3.11	46.84–59.82	69.24 ± 2.34	64.35–74.13	81.71 ± 3.30	74.83–88.60
D	24	51.25 ± 2.32	46.46–56.04	64.83 ± 1.98	60.73–68.93	80.17 ± 2.64	74.71–85.62
E	10	48.50 ± 2.99	41.75–55.25	66.80 ± 3.09	59.81–73.79	78.40 ± 4.93	67.24–89.56
F	5	53.00 ± 7.35	32.60–73.40	64.80 ± 4.85	51.33–78.27	77.80 ± 5.23	63.28–92.32
Total	103	48.35 ± 1.23	45.91–50.79	66.08 ± 1.01	64.08–68.08	80.62 ± 1.24	78.16–83.09

A and B = semen ejaculates collected from the purebred Jersey bull; C–F = semen ejaculates collected from the HF-cross bull. Statistics for proportions of motility (*F* = 4.533, *p*=0.001), live (*F* = 2.127, *p*=0.069), and normal morphology (*F* = 2.148, *p*=0.066).

**Table 3:** One-way ANOVA of semen bacterial count (CFU/ml) among breeding bulls.

Breed	Mean ± SEM	95% CI	*F*	*p* value
Jersey	69.05 ± 32.96	2.48–135.61	0.903	0.344
HF-cross	37.52 ± 15.62	6.28–68.77		
Total	50.38 ± 16.29	18.06–82.69		

CFU/ml = colony-forming units per milliliter.

**Table 4:** One-way ANOVA of bacterial count (CFU/ml) of cryopreserved semen from different ejaculates of bulls.

Ejaculates	No. of straws	Mean ± SEM	95% CI	*F*	*p* value
A	19	141.65 ± 69.50	−4.36–287.66	1.627	0.160
B	24	10.98 ± 8.32	−6.23–28.20		
C	21	23.85 ± 11.32	0.23–47.46		
D	24	52.08 ± 37.77	−26.05–130.22		
E	10	28.20 ± 18.03	−12.60–69.00		
F	5	40.20 ± 24.69	−28.36–108.76		
Total	103	50.38 ± 16.29	18.06–82.69		

A and B = semen ejaculates collected from the purebred Jersey bull; C–F = semen ejaculates collected from the HF-cross bull; CFU/ml = colony-forming units per milliliter.

**Table 5:** Multiple linear regression analysis of semen characteristics and bacterial count of HF-cross and Jersey bulls.

Parameter	Predictors	*B*	SE	*t*	*p*	*R* ^2^	Adjusted *R*^2^	*F*
Motility (%)	Intercept	60.56	11.52	5.26	^*∗∗∗*^	0.37	0.36	29.40
	Bacterial count	−0.05	0.01	−7.67	^*∗∗∗*^			
	Storage time	−0.03	0.04	−0.86	ns			

Live (%)	Intercept	61.49	8.80	6.99	^*∗∗∗*^	0.45	0.44	41.31
	Bacterial count	−0.04	0.01	−8.90	^*∗∗∗*^			
	Storage time	0.02	0.30	0.76	ns			

Normal morphology (%)	Intercept	62.05	10.86	5.71	^*∗∗∗*^	0.45	0.44	41.06
	Bacterial count	−0.05	0.01	−8.56	^*∗∗∗*^			
	Storage time	0.07	0.03	1.95	ns			

SE = standard error of the regression coefficient (*B*); ns = not significant. ^*∗∗∗*^*p* < 0.001.

**Table 6:** Prevalence of bacteriospermia in different ejaculates of HF-cross and Jersey bulls.

Risk factors	Total straws examined	No positive	Prevalence (%)	*χ* ^2^	*p* value
Breed					
Jersey	43	16	37.2	0.082	0.774
HF-cross	60	24	40.0		
Total	103	40	38.8		

Ejaculates					
A	19	13	68.4	18.674	0.002
B	24	3	12.5		
C	21	7	33.3		
D	24	8	33.3		
E	10	5	50.0		
F	5	4	80.0		
Total	103	40	38.8		

A and B = semen ejaculates collected from the purebred Jersey bull; C–F = semen ejaculates collected from the HF-cross bull.

**Table 7:** Logistic regression of bacteriospermia in different ejaculates of HF-crossbred and Jersey bulls.

Predictors	Estimate	SE	*p* value	Odds ratio	95% CI
Intercept	0.773	1.07	0.117		
Breed					
Jersey vs. HF-cross	0.613	2.256	0.616	1.846	0.168–20.26

Ejaculates: A vs.					
B	−2.718	0.052	0.001	0.066	0.014–0.31
C	−2.079	0.151	0.086	0.125	0.012–1.34
D	−2.079	0.150	0.083	0.125	0.012–1.31
E	−1.386	0.321	0.280	0.25	0.020–3.10
F				1	

LR chi^2^ = 19.67; *p* value = 0.0014; *R*^2^ = 0.1430. A and B = semen ejaculates collected from purebred Jersey bulls; C–F = semen ejaculates collected from HF-crossbred bulls; SE = standard error of the estimate/regression coefficient.

## Data Availability

The data used to support the findings of this study are available from the corresponding author upon request.
